# Ratios and Effect Size

**DOI:** 10.1037/xan0000143

**Published:** 2017-08-14

**Authors:** Jasper Robinson

**Affiliations:** 1 School of Psychology, University of Nottingham

**Keywords:** effect size, discrimination learning, discrimination ratio, suppression ratio, reduction

## Abstract

Responding to a related pair of measurements is often expressed as a single discrimination ratio. Authors have used various discrimination ratios; yet, little information exists to guide their choice. A second use of ratios is to correct for the influence of a nuisance variable on the measurement of interest. I examine 4 discrimination ratios using simulated data sets. Three ratios, of the form *a*/(*a* + *b*), *b*/(*a* + *b*), and (*a* − *b*)/(*a* + *b*), introduced distortions to their raw data. The fourth ratio, (*b* − *a*)/*b* largely avoided such distortions and was the most sensitive at detecting statistical differences. Effect size statistics were also often improved with a correction ratio. Gustatory sensory preconditioning experiments involved measurement of rats’ sucrose and saline consumption; these flavors served as either a target flavor or a control flavor and were counterbalanced across rats. However, sensory preconditioning was often masked by a bias for sucrose over saline. Sucrose and saline consumption scores were multiplied by the ratio of the overall consumption to the consumption of that flavor alone, which corrected the bias. The general utility of discrimination and correction ratios for data treatment is discussed.

I examine the use of two methods for treating data to maximize statistical sensitivity: transforming data *into* a discrimination ratio, and treating data *with* a ratio that corrects for the influence of an unwanted variable. It is generally useful to apply a transformation to data (e.g., Howell, 2002). This may be to better meet assumptions for parametric analysis (e.g., log transformation of negatively skewed latency data; see, e.g., Miller, Laborda, Polack, & Miguez, 2015). A different motive is to improve statistical sensitivity. Discrimination ratios (e.g., *a*/(*a* + *b*), Kamin, 1969), see below for full description) offer two important benefits: In addition to simplifying analysis by converting a pair of raw numbers (e.g., instrumental response rates during conditioned stimulus and baseline measurements) into a single ratio, the discrimination ratio can reduce subject-by-subject variability because of its accommodation of baseline (*b*) response rates. It is this second feature of the discrimination ratio that offers improved statistical sensitivity. Rather little is known about discrimination ratios’ properties. To this end, I describe analyses that use synthetic data to characterize the effects of discrimination ratios on data and especially on their statistical sensitivity. In the second section of this report I describe empirical data whose effect of interest is masked by the influence of the specific stimuli used. In these sensory preconditioning experiments, rats’ preference for a control flavor over one with aversive properties was, in many experiments, masked by an overriding preference for sucrose over saline. Sucrose and saline were counterbalanced to serve as either the control or the aversive flavor. I describe a simple method for correcting for the intrinsic sucrose-saline bias seen in such experiments and examine its effects on statistical sensitivity.

## Data Treatment

For analyses of both discrimination ratios and the correction ratio, standard parametric analyses were used for null-hypothesis testing. Tests evaluated two-tailed hypotheses and α = .050. Partial eta squared (η_p_^2^) was used to represent main effect and interaction effect sizes. Standardized 90% confidence intervals (CIs) for η_p_^2^ were computed using the methods described by Kelley (2007) and used his MBESS package for R (Version 3.3.2. [Computer software], Vienna, Austria). Bayesian analyses supplemented the interpretation of a key results (JASP (Version 0.8 Beta 5) [Computer software]. Amsterdam, The Netherlands). The Bayes factor (BF) specifies the ratio of the probabilities between a target model (BF10) and an appropriate comparison, such as the null model (BF01). The magnitude of the ratio is taken to reflect the likelihood of the support for the target model, which may be instructive in interpreting data. Jeffreys (1961, as cited in, Rouder, Speckman, Sun, Morey, & Iverson, 2009) maintained that BFs greater than 3 may be considered “some evidence” for one hypothesis over its alternative hypothesis, with BFs of 10 or more or 30 or more as, respectively, “strong” and “very strong” evidence.

## Four Discrimination Ratios

Kamin (1969) used the discrimination ratio, *a*/(*a* + *b*), in conditioned suppression experiments. When *a* is nonnegative and *b* is greater than zero, the ratio will vary between 0 and 1.0 with 0.5 corresponding to *a* and *b* having equivalent values. In a conditioned suppression experiment, *a* represents the instrumental response rate (e.g., Bonardi & Jennings, 2009; Robinson, Whitt, Horsley, & Jones, 2010) or lick rate (e.g., Pezze, Marshall, & Cassaday, 2016) during a conditioned stimulus for shock (conditional stimulus [CS] rate); and *b* represents a baseline response rate (e.g., the instrumental or lick rate immediately before the presentation of the conditioned stimulus; Pre-CS rate). Here, similar CS and Pre-CS rates will yield ratios that approximate 0.5. They will approach zero as responding to the conditioned stimulus becomes suppressed, for example during the acquisition of the conditioned response. Another purpose is to simplify the performance of birds in an appetitive discrimination (e.g., George & Pearce, 1999). Here *a* and *b* might be the response rates of, respectively, food reinforced and nonreinforced stimuli. Successful discrimination is reflected in *a*’s values exceeding *b*’s and in discrimination ratios rising from chance (0.5) to approach 1.0 (see also, Harris, Shand, Carroll, & Westbrook, 2004; Montuori & Honey, 2016).

Other ratios are possible that capture the discrimination between a pair of *a* and *b* values and I will describe three that have been used in experimental psychology. Redhead has reported data from an appetitive discrimination with pigeons in which *a* and *b* refer, respectively, food-reinforced and nonreinforced conditioned stimuli (Redhead & Curtis, 2013; Redhead & Pearce, 1998). They used the ratio *b*/(*a* + *b*) to capture each bird’s discrimination. Birds’ performance began at around 0.5 and progressed toward 0 as responding became focused on the food-reinforced trials, represented by *a*. A third ratio was used by Ennaceur and Delacour (1988) to summarize discrimination of rats’ exploration of novel (*a*) and familiar (*b*) junk objects in recognition memory experiments. Their ratio has the form (*a* − *b*)/(*a* + *b*). Rats’ biased their exploration toward the novel object, represented by *a*, giving positive Ennaceur ratios (i.e., 1 ≥ ratio >0). Notice that the three ratios’ share their denominator but differ in their numerator. The fourth ratio that I will consider has a different denominator and the form: (*b* − *a*)/*b*. This ratio was used by Pfautz, Donegan, and Wagner (1978; see also Hoffman, Selekman, & Fleshler, 1966) in Pavlovian shock conditioning experiments with rats and rabbits. *a* and *b* refer, respectively, to the response rates (lever pressing or heart rate) during the conditioned stimulus and to the baseline rate. Pfautz ratios are zero when *a* is equivalent to *b* (e.g., before conditioning has taken place) and approach one as responding is suppressed during the conditioned stimulus. All four of these ratios have the advantage over a simple ratio (e.g., *a*/*b*) that they will be bound within a fixed range of values. The properties of these four ratios were characterized by systematically generating data sets and comparing them to one another. These simulations were intended to help to understand potential distortions that each ratio produces from the primary data and to assess potential differences in their statistical sensitivity.

### Surface Plots of the Four Discrimination Ratios

**Figure 1 fig1:**
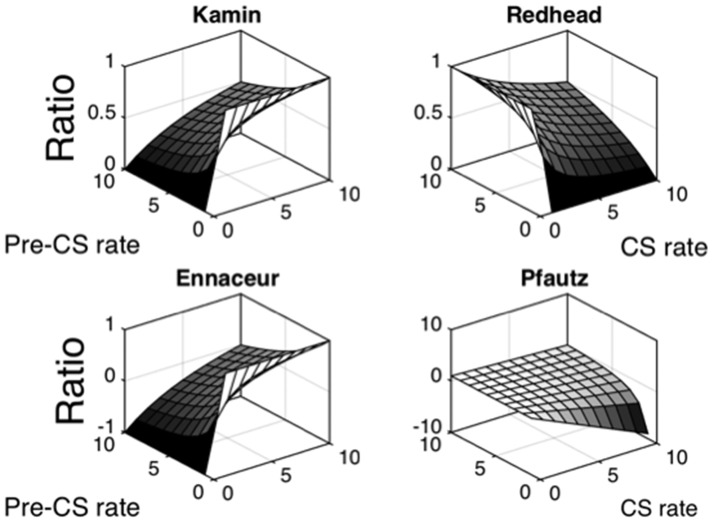
Surface plot showing the relationships between varied conditional stimulus (CS) rates and Pre-CS rates and four different discrimination ratios. Each surface plot depicts the ratios that are computed by the systematic variation of CS rates and Pre-CS rates varied between 1 and 10 in one-unit intervals.

The top left surface plot of Figure 1 displays the relationship between *a* (e.g., CS) and *b* (Pre-CS) rates and Kamin ratios using hypothetical data. The Matlab code and figure are included in the supplemental materials. The Matlab figure allows rotation of the surface plots and specification of the axis values to facilitate inspection. The ordinate indicates the Kamin ratios that are derived when Pre-CS and CS rates are each varied between 0 and 10 in one-step intervals. The rear left panel of the surface plot indicates data derived when CS rates do not exceed Pre-CS rates, as in a conditioned suppression experiment (e.g., Bonardi & Jennings, 2009; Robinson et al., 2010); the rear right panel of the surface plot indicates data derived when *a* rates do exceed *b* rates (cf., George & Pearce, 1999). Kamin ratios will approach 0 as CS rates approach zero and 1.0 as Pre-CS rates approach 0. Despite the one-step intervals between each CS and Pre-CS response rate being linear, their relationship to the Kamin ratio is nonlinear. In particular, the relationship bows as the Pre-CS and CS rates reach parity (i.e., where the Kamin ratio equals = 0.5).[Fig-anchor fig1]

The top right, and lower pair of surface plots in Figure 1 demonstrate the relationship between *a* and *b* rates using, the Redhead (Redhead & Curtis, 2013; Redhead & Pearce, 1998), Ennaceur (e.g., Ennaceur & Delacour, 1988), and Pfautz (Pfautz et al., 1978) ratios. The Ennaceur ratio produces an identically shaped plot to the Kamin ratio, albeit with a different range of ratio values. The Redhead ratio produces a plot having the mirror image of the Kamin ratio plot and has the same range of values. The Ennaceur ratio will yield ratios approaching minus one in conditioned suppression experiment where Pre-CS ratios (*b*) exceed CS ratio (*a*; e.g., Robinson, Sanderson, Aggleton, & Jenkins, 2009). At parity the rates will give a ratio of zero and when the *a* rate exceeds the *b* rate Ennaceur ratios approach positive one (e.g., Ennaceur & Delacour, 1988; Whitt, Haselgrove, & Robinson, 2012; Whitt & Robinson, 2013). The Redhead ratio will be zero with *a* and *b* rate parity and will mirror the Kamin ratio both in typical conditioned suppression ratio experiments (i.e., ratios approach one rather than zero during suppression) and in appetitive discrimination experiments (i.e., ratios approach zero rather than one on master of the discrimination; e.g., Redhead & Curtis, 2013; Redhead & Pearce, 1998). The Pfautz ratio’s surface plot is different from those of the other three ratios. Although the ratio’s surface plot becomes nonlinear when Pre-CS rates are low and CS rates are high (i.e., the bottom right region of the surface plot’s box), elsewhere it retains much more of the linearity of the CS and Pre-CS rates (note that this linearity is more evidence in Figure 3, which is discussed below).

### Comparison of Effect Sizes From Kamin and Pfautz Ratios

The previous examination of the Kamin, Ennaceur, and Redhead ratios indicated that, although the specific values of the ratios differed, they behaved similarly in their representation of CS and Pre-CS rates. In particular, the ratios’ surface plots and the effect sizes of their one-sample-*t* statistics were similar. Because of that similarity, the current analysis considers only one of those three (the Kamin ratio), and compares it to the Pfautz ratio, whose characteristics are different.

#### Simulations methods

R (Version 3.3.2. [Computer software], Vienna, Austria) was used to generate 500 normally distributed data points that varied around a mean of 1 and had a *SD* of 0.1. These were to serve as the *a* values in a population of 500 Kamin ratios. The code is included in the supplemental materials. The *a* distribution generation was initiated using the “seed” number 1. Simulations using the same seed produced the same distribution, allowing identical simulations to be created when needed. A second and third distribution was created using the same process and the same seed number but the *SD*s were increased to 0.2 and to 0.3. The process for the generation of a trio of *a* distributions with means of 1 was repeated for distributions with means of 8, 15, 22, 29, 36, and 43; thus, being equally spaced and symmetrical with respect to the midpoint, 22. These steps created a series of 18 *a*-distributions with three different standard distributions, six different means and the same seed value, 1. The process was repeated with new seeds taken from the natural integer series: 2, 3, 4. . . . To prevent the subsequent generation of unusual ratios (i.e., >1 and <0), normal distributions that generated negative values were not used. Eight seeds were used in total and these processes yielded 144 sets of normally distributed data (i.e., eight seeds × six means × 3 *SD*s).

The process for generation of Kamin ratios was repeated for the Pfautz ratios. The same seeds were used to permit meaningful comparison of the ratios that were generated.

Next all data were used to compute Kamin and Pfautz ratios with a fixed *b* value of 22, that is, the midpoint on the *a* series. Except for ratios based on *a* distributions with a mean of 22, one-sample *t*, and associated, statistics were calculated for the ratios, with the Kamin ratios being compared with μ = 0.5 and the Pfautz ratio being compared with μ = 0.0. The statistics were used to examine possible variation in the level of sensitivity to detect differences from μ across the profile of ratios.

#### Simulation results

**Figure 2 fig2:**
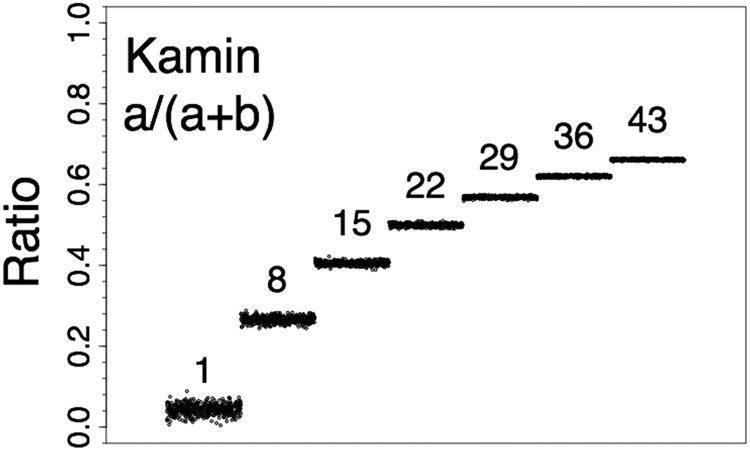
Discrimination ratios for simulated data sets computed by Kamin’s method. Computer generated data with *SD*s of 0.3 and means of either: 1, 8, 15, 22, 29, 36, or 43 (*n*s = 500) were used to compute discrimination ratios of the form *a*/(*a* +*b*). Here, *a* (the conditional stimulus [CS] rate) corresponds to the generated data and *b* (the Pre-CS rate) was 22 for all ratios.

Two seeds in the natural integer sequence, 1–10, yielded *a*-distributions that were discarded because their seed created one or more negative values. This left eight *a*-distributions that contained no negative values. An example of Kamin-ratio data based on *a*-distributions having a *SD* of .3 is given in Figure 2. As the mean values increased across the series (i.e., 1, 8, 15, 22, 29, 36, and 43) the ratios increased, a pattern that may be likened to the extinction of conditioned suppression (e.g., Ward-Robinson & Hall, 1999). Notice that the Kamin ratios increase nonlinearly and cluster in the region where *a* becomes equivalent to *b*, just as Figure 1 depicts. Or, as an alternative view, the pairs of mean *a* rates 1 and 43, 8 and 36, and 15 and 29 are equivalently distant from the *b* rate, 22, but their ratios are not equidistant. A third feature is that the lower the mean value of the distribution, the greater the variability of the Kamin ratios. The Figure 2 data correspond to the Kamin surface-plot in Figure 1. In particular, the Figure 2 data correspond to the ratios on the back surfaces of the surface plot (most of the left side and a smaller portion of the right side nearest the corner). The code for the generating the ratios is available in the supplemental materials.[Fig-anchor fig2]

**Figure 3 fig3:**
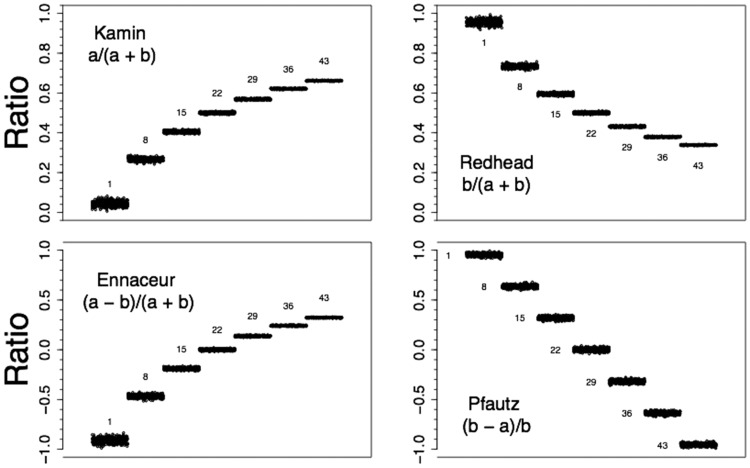
Discrimination ratios for simulated data sets computed by four different methods. Computed generated data with *SD*s of 0.3 and means of either: 1, 8, 15, 22, 29, 36, or 43 (*n*s + 500) were used to compute discrimination ratios of the form *a*/(*a* + *b*), *b*/(*a* + *b*), (*a* − *b*)/(*a* + *b*), and (*b* − *a*)/*b*. Here, *a* (the conditional stimulus [CS] rate corresponds to the generated data and *b* (the Pre-CS rate) was 22 for all ratios.

Example data from all four ratios are presented in Figure 3. Example code is given in the supplemental materials. The ratio data are from simulations with the *SD* of .3 and, from right to left, indicate ratios that might be found during extinction of conditioned suppression (e.g., Ward-Robinson & Hall, 1999). All data in Figure 3 were computed based on distributions having the same random seed number. The Ennaceur ratio produced a similar distribution of ratios as the Kamin ratio, albeit with values on a different scale; that is: (a) The linear *a*-rates produced nonlinear ratios; and (b) There was greater variability in ratios associated with lower *a*-rates. The Redhead and Pfautz ratio declined in value as the *a* values increased. The Redhead ratio produced a similar nonlinear profile as the Kamin and Ennaceur ratios and, likewise, had greater variability in ratios associated with lower *a*-rates. As was seen in Figure 1, the Pfautz ratio differs from the other three ratios in that the linear sequence in the *a* rates is retained in its ratios. Another difference is that the variability is similar across ratios computed from data with all levels of *a* rate. This description of the data was supported by linear-regression analysis: The Pfautz data in Figure 3 were perfectly described as linear trends, *R*^2^ = 1.000; the remaining three ratios’ data were more accurately described as cubic trends, 0.996 ≤ *R*^2^ ≤ 0.999, than linear, 0.910 ≤ *R*^2^ ≤ 0.990, or quadratic trends, 0.993 ≤ *R*^2^ ≤ 0.996.[Fig-anchor fig3]

Table 1 gives further information about the properties of the four types of ratio. Its upper panel simply gives the ratios for each of the *a* rates (CS rate) with a *b* rate (Pre-CS rate) of 22. The ratio thus approximates the simulated data in Figure 3. Comparison is made of the seven ratios of each type to its μ value. Mu is taken as the ratio value where CS and Pre-CS rates are both 22. Comparison is made using one-sample *t* tests and associated *p* and effect sizes are given. The average of the seven ratios differs across the four ratios but the absolute difference between the mean ratio and μ is the same for the Kamin and Redhead ratios. The Kamin, Redhead, and Ennaceur ratios share *t*, *p*, and effect size statistics. The Pfautz ratio stands alone in this comparison: With the rates used here, the ratios are more sensitive in that the one-sample *t* was better able to detect a difference from μ.[Table-anchor tbl1]

#### Analysis of effect sizes from kamin and pfautz ratios

**Figure 4 fig4:**
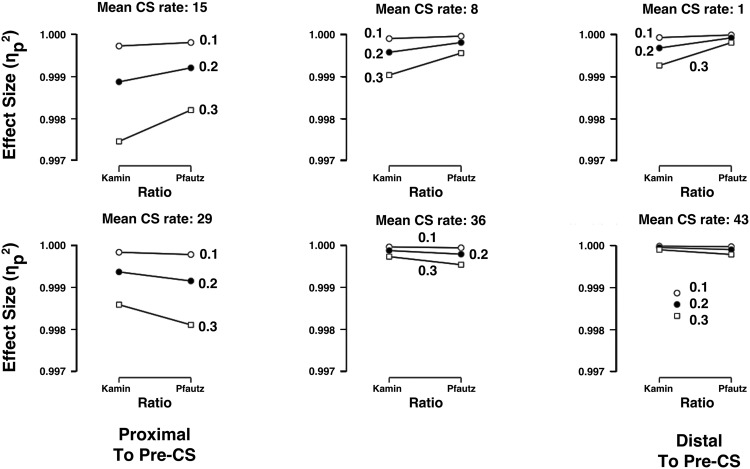
Mean effect sizes for one-sample *t*s that compare simulated discrimination ratios to μ ratios. Computer generated data with *SD*s of 0.1, 0.2, and 0.3 and means of either:1, 8, 15, 22, 29, 36, or 43 (*n*s = 500) were used to compute discrimination Kamin [*a*/(*a* + *b*)] and Pfautz [(*b* − *a*)/*b*] ratios. Here *a* (the conditional stimulus [CS] rate) corresponds to the generated data and b (the Pre-CS rate) was 22 for all ratios. Each of the 36 ratios was compared with μ, which was the .5 for the Kamin ratios and 0 for the Pfautz ratio, using one-sample *t* tests. The six graphs’ ordinates summarize effect-size statistics (η_p_^2^) to give indication of the sensitivity of each method under the varied conditions. The effect sizes are generally high because of the relatively large sample sizes. Error bars depicting 90% confidence intervals are obscured by the figure symbols.

The Kamin and Pfautz ratios that were generated above were evaluated by reference to their respective μs (i.e., 0.5 for the Kamin ratio and 0 for the Pfautz ratio) using one-sample *t* tests whose effect size statistics are summarized in Figure 4. Raw data and statistical analysis are supplied in the supplemental materials. The simulated data had small *SD*s and large *n*s (*n* = 500), which produced large effect sizes. The data show that effect sizes were, unsurprisingly, larger from ratios based on smaller *SD*s. Ratios that were based on CS rates that were proximal to the Pre-CS rate, 22, (i.e., from distributions with average *a* rates of 15 or 29) were lower than ratios based on *a* rates further from 22, especially in combination with larger *SD*s. This is especially clear in the ratios whose CS rates averaged 1 and 43, with rates of 8 and 36 being in-between the two extremes. Most significant was the variation in the effect sizes of the Kamin and Pfautz ratios. The top row summarizes data that correspond to conditioned suppression, that is, where the mean CS rates (15, 8, and 1) are lower than the Pre-CS rate. Here, the Pfautz ratio appeared to produce superior effect sizes to the Kamin ratio. The reverse appeared to be the case in elevated ratios, those summarized in the lower row with mean CS rates are higher than the Pre-CS rate, 22.[Fig-anchor fig4]

**Figure 5 fig5:**
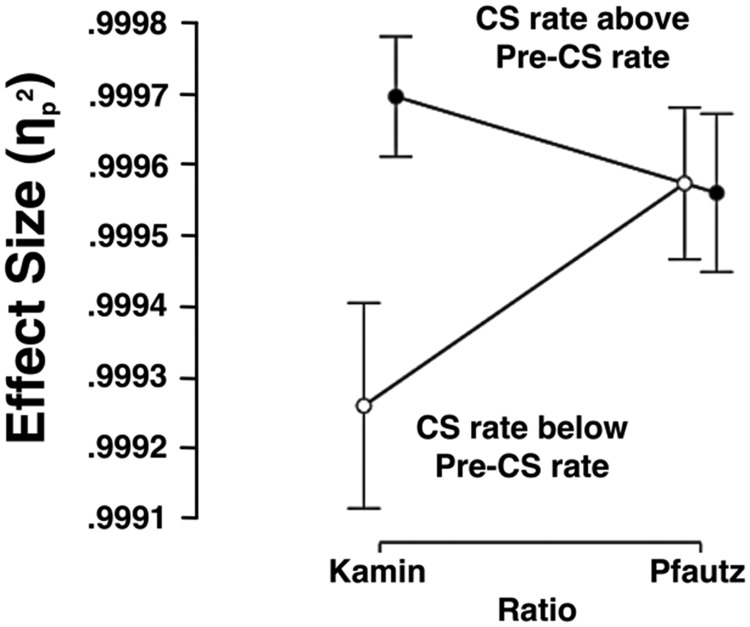
A representation of the data summarized in Figure 4 that is pooled over the 3 *SD*s and the six varied conditional stimulus [CS]-rates. Mean effect sizes for one-sample *t*s that compare simulated discrimination ratios to μ ratios are presented. Computed generated data with *SD*s of 0.1, 0.2, and 0.3 and means of either: 1, 8, 15, 22, 29, 36, or 43 (*n*s = 500) were used to compute discrimination Kamin [*a*/(*a* + *b*)] and Pfautz [(*b* − *a*)/*b*] ratios. Data are pooled over the *SD*s and are coded as either above (i.e., 22, 29, and 43) the Pre-CS rate (22) or below the Pre-CS rate (i.e., 1, 8, and 15). Here, *a* (the CS rate) corresponds to the generated data and *b* (the Pre-CS rate) was 22 for all ratios. Each of the 36 ratios was compared with μ, which was the .5 for the Kamin ratios and 0 for the Pfautz ratio, using one-sample *t* tests. The graphs ordinate summarizes effect-size statistics (η_p_^2^) to give indication of the sensitivity of each method under the varied conditions. The effect sizes are generally high because of the relatively large sample sizes. Error bars represent 90% confidence intervals.

To simplify and focus the main features of the simulated ratio data, they were recoded with the *SD* variable omitted and with the mean-CS-rate variable recoded more crudely as being suppressed or elevated (i.e., either above or below the Pre-CS rate of 22). These simplified data are summarized in Figure 5. The effect sizes from Kamin ratios were higher when they were based on elevated CS rates than on suppressed CS rates. By contrast, the Pfautz ratios’ effect sizes appeared unaffected by their side of the Pre-CS. This description of the data was supported by ANOVA (analysis of variance), which did not detect a main effect of the Kamin versus Pfautz ratios, *F*(1, 284) = 1.6; *p* > .196; η_p_^2^ < .007, 90% CI [.000, .029]. However, the ANOVA detected a main effect of suppression versus elevation, *F*(1, 284) = 9.5; *p* < .003; η_p_^2^ > .031, 90% CI [.007, .072], and a Kamin/Pfautz × Suppression/Elevation interaction, *F*(1, 284) = 10.8; *p* < .002; η_p_^2^ > .036, 90% CI [.009, .078]. The source of this interaction was investigated with simple main effects analyses, using the common-error term. The effect size difference in elevated Kamin and Pfautz ratios was unreliable, *F*(1, 284) = 2.0; *p* > .161; η_p_^2^ < .006, 90% CI [.000, .031], but the corresponding difference for suppressed ratios was reliable, *F*(1, 284) = 10.5; *p* < .002; η_p_^2^ > .035, 90% CI [.008, .076]. Elevated Kamin ratios were reliably higher than suppressed Kamin ratios, *F*(1, 284) = 20.3; *p* < .001; η_p_^2^ > .066, 90% CI [.026, .116], but no such elevation/suppression difference was detected for Pfautz ratios, *F* < 1; *p* > .89; η_p_^2^ < .001, 90% CI [.000, .005]. A Bayesian ANOVA was performed with models corresponding to the previous ANOVAs variables and matched its findings. The model based on the Kamin/Pfautz × Suppression/Elevation interaction was strongly favored over the combined Kamin/Pfautz and Suppression/Elevation models, BF10 > 23.7. These analyses are included in the supplemental materials.[Fig-anchor fig5]

This analysis indicated that the Pfautz ratio behaved similarly to suppressed and to elevated CS rates: Effect size statistics associated with one-sample *t*s were indistinguishable by inferential testing. By contrast the Kamin ratio produced greater effect size statistics with elevated data than with suppressed data. It is notable that the absolute differences in the effect sizes are trivially small and might lead one to conclude that all of the ratios produce excellent effect sizes. However, the synthetic data used here have large sample sizes and this will boost effect size statistics to points beyond those typically seen in empirically obtained data. Furthermore, because η_p_^2^ will not exceed 1 the absolute differences in these synthetic data are likely to be compressed. Thus, the absolute difference in the effect size statistics in empirical data is likely to be greater than that seen here.

### Discussion

The Kamin (1969), Redhead (Redhead & Curtis, 2013; Redhead & Pearce, 1998), and Ennaceur (e.g., Ennaceur & Delacour, 1988) ratios produced similar distortions on the simulated conditioned suppression data. Ratios based on low CS rate (*a*) had greater variability than CS rates that were similar to the Pre-CS rate (*b*). Furthermore, the space between the ratios corresponding to neighboring CS rates was uneven: Rather than corresponding to the equal steps between each CS rate, they were relatively compressed as the CS rate approximated the Pre-CS rate. The Pfautz ratio (Pfautz et al., 1978) suffered neither of those complications: Ratios for different CS rates did not differ in their variability and the interval between each set of ratios retained the linearity of the original CS rates.

The Kamin and Pfautz ratios differed in their sensitivity as measured by effect size statistics based on one-sample *t* tests that compared each CS-rate’s population of ratios to the value of the ratio when the *a* and *b* rates were equal. In particular, the Kamin ratio suffered a marked loss in effect size when the *a*-rate was far lower than the *b*-rate, the situation in conditioned suppression experiments. The implication of this is that we should not use Kamin’s ratio for conditioned suppression experiments, or any other procedure in which the aim is to detect effects when *a* < *b*. Instead, we should favor Pfautz’ ratio. The Kamin ratio has been favored in conditioned suppression experiments for the last five decades and these new findings indicate that effect sizes may have been underestimated.

The Kamin ratio produced better effect sizes when the CS rate (*a*) exceeded the Pre-CS rate (*b*). This arrangement is often seen in experiments where the *a*-rate rises during mastery of a discrimination and the *b*-rate may either decline or remain an estimate of a constant baseline rate (e.g., George & Pearce, 1999; Harris et al., 2004; Montuori & Honey, 2016). The implication of these simulations is that the Kamin ratio is a suitably sensitive treatment for such data. Although there was no inferential statistical support for the observation, the mean value for the Kamin ratio when *a* > *b* was the largest of the four ratios. The Pfautz ratio’s effect sizes were indistinguishable when applied to data of either form (i.e., either *a* < *b* or *a* > *b*). The natural conclusion from these observations is that the Pfautz ratio should be used by default: It does not distort its input data and produces robust effect sizes that are equal for both *a* < *b* or *a* > *b* data.

I emphasize that these conclusions are based on very large sets of synthetic data that may detect ratio differences in effect size that would be rendered marginal in real experimental data with smaller *n*s. Nevertheless, researchers are encouraged to report effect sizes, not only for their own individual experiment, but to allow aggregated effect sizes to be computed that are based on *many* similar experiments (e.g., Cumming, 2011; Lakens, 2013). Thus, even small differences in the effect sizes from particular ratios may ultimately become important.

Skewed data sets could benefit from the distorting influence of some of these ratios. Consider lick-suppression, latency data (e.g., Miller et al., 2015; Pezze et al., 2016) that will often be negatively skewed: They will be relatively diffuse at long latencies and compressed at short latencies, as they approach the floor of zero seconds. This pattern of compression and expansion is the complement of the distortions appreciable in Figure 3 seen for the Kamin, Redhead, and Ennaceur ratios. Thus, depending upon the level of responding at which key effects are to be detected, these ratios could outperform the Pfautz ratio with negatively skewed data.

## A Correction Ratio to Eliminate the Influence of a Nuisance Variable on Effect Size

The motive for applying the discrimination ratios above is to reduce data variability to better support statistical analysis. The discrimination ratios achieve this by compensating for subject-by-subject variation in one variable (e.g., Pre-CS rate) to allow more sensitive data analysis of the target variable (e.g., CS rate). I now describe a second ratio-based technique to reduce variance to improve data sensitivity. Rather than operate at subject-by-subject variability, this method applies a correction ratio to offset distortions produced by nuisance variables. I exemplify this with an example from a gustatory sensory preconditioning procedure in which the nuisance variable is based on intrinsic differences in rats’ consumption of two flavored solutions. This interferes with detection of differences in consumption based on the experimental treatment. The correction ratio technique is quite general and broader applications will be considered.

### An Application of the Correction Ratio to Sensory Preconditioning

Rescorla and Cunningham (1978) reported within-subject sensory preconditioning data with rats. Their procedure involved rats first receiving a pair of compound flavors on separate trials (e.g., sucrose-acid and saline-quinine). To reveal learning about the co-occurrence of each pair of flavors, one flavor (e.g., acid) was paired with illness to create an aversion to it. Rescorla and Cunningham reported a marked reduction in consumption of the flavor whose partner was illness-paired (i.e., sucrose in this example). The experiment was counterbalanced such that for half of the rats, sucrose was made aversive and saline was the control flavor and for the remaining rats saline was aversive and sucrose was the control flavor. Although successful in demonstrating sensory preconditioning, there was a pronounced overall preference for sucrose over saline during testing. This preference may have acted against Rescorla and Cunningham detecting sensory preconditioning (see also Ward-Robinson, Symonds, & Hall, 1998).

Unpublished data from a similar sensory preconditioning procedure are presented in Table 2. Uncorrected fluid consumption data, measured in grams, are displayed in the left side of the upper panel with summary statistics below. Data in columns headed ‘A+’ refer to the flavor whose consumption is expected to be low because its partner had been paired with illness. Data headed ‘B−’ refer to the control flavor whose consumption should be higher than A+’s. The procedure reliably biased rats’ consumption toward B− (19.2 g) relative to A+ (8.2 g), *t*(16) = 3.1, *p* < .007, η_p_^2^ > .379, 90% CI [.01, .46]; that is, sensory preconditioning was obtained. However, this difference was obtained despite a twofold bias in the consumption of S (18.4 g) over *N* (9.1 g), *t*(16) = 2.4, *p* < .027, η_p_^2^ > .273, 90% CI [.12, .59].[Table-anchor tbl2]

This unwanted flavor bias was corrected by multiplying each uncorrected sucrose score by the ratio of the overall mean consumption and the uncorrected sucrose score, irrespective of its role as A+ or B−. Thus, the rat in the first row’s uncorrected sucrose score of 2 g reduced to 1.5 g to accommodate the fact that sucrose consumption was generally high. The correction is arrived at because (13.71/18.35) * 2 g ≈ 0.75 * 2 g ≈ 1.5 g. The same process applied to that rat’s saline score increased it from 23 to 34.8 g to reflect saline’s generally low consumption. The correction is (13.71/9.06) * 23 g ≈ 1.51 * 23 g ≈ 34.8 g. The application of these two correction ratios to all the original, uncorrected data produced a complete set of corrected data in which the overall consumption of sucrose is matched with that of saline. The correction treatment also exaggerated discrimination, which is reflected in the means A+ (7.5 g) and B− (19.9 g), and greater effect-size *t*(16) = 3.9, *p* < .002, η_p_^2^ > .492, 90% CI [.42, .77].

**Figure 6 fig6:**
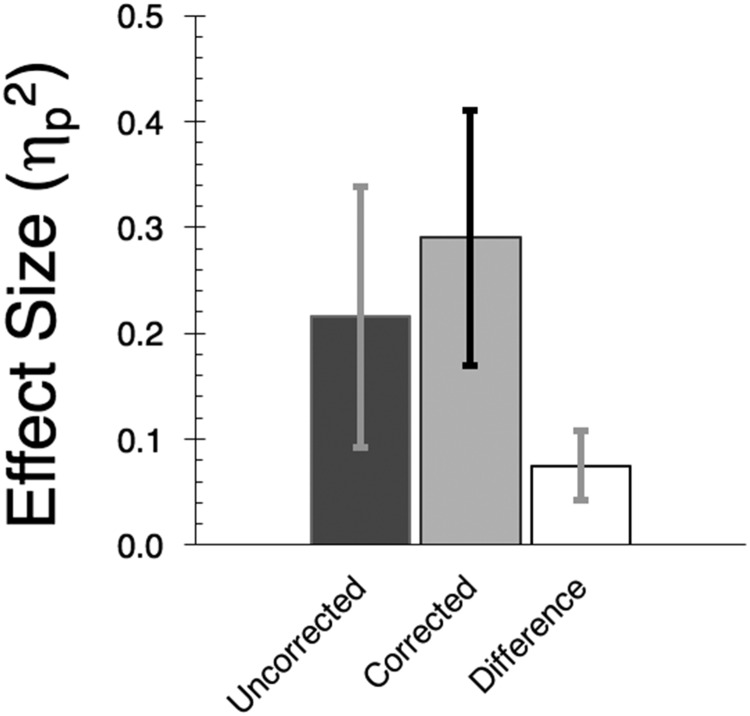
Mean effect sizes (η_p_^2^) for 11 sensory preconditioning experiments using sucrose or saline as test flavors. Data are in their original form (“Uncorrected”) or when subject to the correction treatment (“Corrected”). The third column summarizes values of the corrected minus the uncorrected effect size statistics. Error bars represent 90% confidence intervals.

An additional 10 flavor, sensory preconditioning tests were subjected to this correction treatment and the effects on effect size and sample requirement examined. Some data came from unpublished observations; others came from published data (Ward-Robinson, Coutureau, Good, Honey, Killcross, & Oswald, 2001; Ward-Robinson, Coutureau, Honey, & Killcross, 2005; Ward-Robinson et. al., 1998; Ward-Robinson, Wilton, Muir, Honey, Vann, & Aggleton, 2002). Figure 6 summarizes changes in the effect sizes and in the sample requirements of these experiments when data were in their original, uncorrected form and in their corrected form. The raw data are available in the supplemental materials. Although the effect size statistics were quite variable there was an apparent increase when the correction method was applied, *t*(10) = 4.1, *p* < .003, η_p_^2^ > .625, 90% CI [.21, .76].[Fig-anchor fig6]

**Figure 7 fig7:**
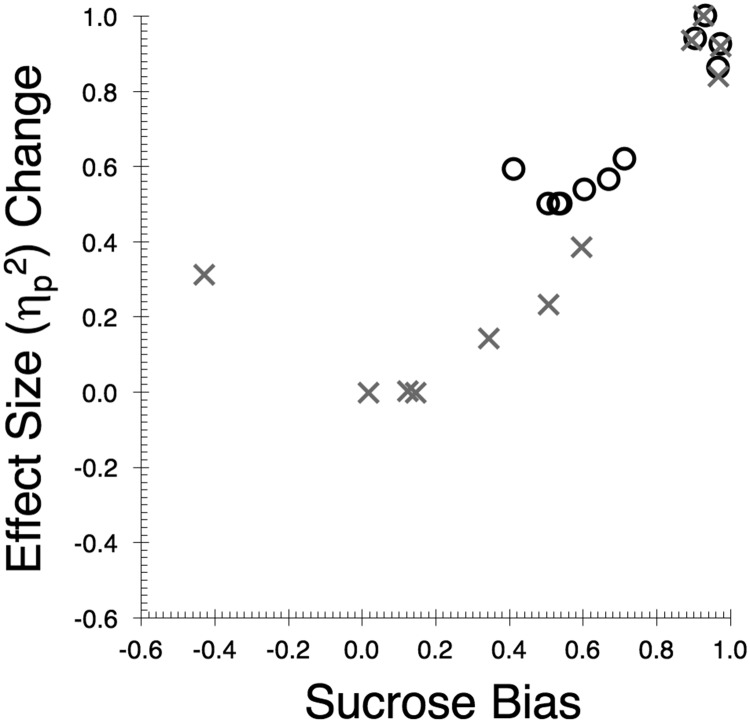
Relationship between the level of the bias in sucrose/saline consumption (abscissa) and the effect of the correction ratio on the sensory preconditioning effect size (ordinate) in the 11 experiments. The Kamin method was used for data represented by circular symbols: Flavor bias was captured using the ratio *S*/(*S* + *N*), where *S* and *N*, respectively, refer to sucrose and saline consumption. The change in effect size of correction ratio took the form, *C*/(*C* + *U*), where *C* and *U* stand, respectively, for the effect sizes of the corrected and uncorrected data. The cross symbols represent the same data but transformed with the Pfautz methods; that is, for flavor using (*S* - *N*)/*S* and for effect size change with (*U* − *C*)/*C*.

The sucrose preference that is represented in Table 2 was not universal in the full set of 11 observations: In some tests there was a marked preference for sucrose over saline; in other tests it was negligible (i.e., in cases where consumption of sucrose and saline was well matched). The sucrose/saline bias across the 11 tests is summarized in Figure 7 and the raw data are given in the supplemental materials. The Kamin and Pfautz methods were each used to express the bias for sucrose over saline on the abscissa. They, respectively, used the ratios S/(S + N) and (S − N)/S where S refers to the overall sucrose consumption, irrespective in its role as A+ or B−, and *N* refers to the corresponding data for saline. The effect sizes of the sensory preconditioning effect (i.e., the difference in consumption between A+ and B−) were approximated with η_p_^2^ for each experiment in its uncorrected (U) and corrected (C) forms. The change resulting from the correction was captured using the Kamin and Pfautz methods using the ratios C/(C + U) and (U − C)/U, respectively. It is apparent that in experiments with little evidence of a preference the benefit of the correction ratio on sensory preconditioning’s effect size was absent. Furthermore, the benefit of using the correction ratio increased the more extreme the flavor bias became. Pearson’s Product Moment Correlation Coefficients, supported that description of the relationship for both the Kamin method, *r*(10) = +.91, *p* < .001, and for the Pfautz method, *r*(10) = +.81, *p* < .001.[Fig-anchor fig7]

### Discussion

In general, the correction ratio offset the unwanted bias in flavor preference and improved the sensory preconditioning effect-size. The sensory preconditioning experiments varied in the extent of the flavor bias: In some there was a marked preference for sucrose over saline but in others there was none. The improvement in effect size was commensurate with the magnitude of the flavor bias: In experiments with large flavor biases, the correction ratio gave a correspondingly large improvement in the sensory preconditioning effect size; when there was no marked flavor bias, the ratio had no appreciably influence on the sensory preconditioning effect size.

The correction ratio also resolves a problem affecting the decision to use stimuli from the same or from different modalities in discrimination tasks. One could assist discrimination by selecting stimuli from different modalities (e.g., a tone and a light in an appetitive discrimination with rats). However, such perceptually distinct stimuli often elicit different patterns of unconditioned response that differ in modifying the measured response (e.g., Jacobs & LoLordo, 1977) and may encourage selection in intramodal stimuli (e.g., a tone and a clicker). One solution is, thus, to facilitate discrimination by the selection of stimuli from different modalities before offsetting unwanted variation with the correction ratio.

The sensory preconditioning examples summarize here were taken from within-subjects experiments in which fluid consumption was measured. Of course, this correction ratio could be applied elsewhere to different experimental procedures with alternative stimuli and measurement variables. For example, George and Pearce (1999) reported an experiment that suffered from an unwanted difference in the discriminability of two types of counterbalanced stimuli. In other regards, their experiment was quite different from the sensory preconditioning experiments: It used a between-subjects design, an autoshaping procedure with pigeons and a Kamin discrimination-ratio dependent variable, which was based on peck rates during reinforced and nonreinforced keylight stimuli. Two groups of pigeons received either an intradimensional shift or an extradimensional shift. The dimensions were colors and orientations, which were combined as keylight stimuli. For some of the intradimensional shift pigeons color was relevant to reinforcement and orientation was irrelevant; for the remainder, orientation was relevant and color irrelevant to reinforcement. The same counterbalancing arrangement was applied to the extradimensional shift pigeons to permit meaningful comparison of the performance across intra-/extradimensional shifts. George and Pearce’s intradimensional pigeons out-performed the extradimensional pigeons but that effect was masked by the tendency in the two subgroups within each main group to learn more quickly if their relevant dimension was color, rather than orientation (see also, Mackintosh & Little, 1969; Urcuioli & Zentall, 1986).

Despite these differences from the sensory preconditioning procedure examined above, the correction ratio may be applied in the same way to George and Pearce’s (1999) data. For example, by the fifth session that George and Pearce present in their Figure 2, intra- and extradimensional ratios are, respectively, 0.984 and 0.933 in pigeons whose relevant dimension was color and 0.822 and 0.690 in pigeons whose relevant dimension was orientation. The bias in discrimination between color and orientation can be offset in the same way as for sensory preconditioning by the multiplication of each discrimination ratio by the appropriate correction ratio. The denominator for color correction ratio will be the average color discrimination ratio (i.e., (0.983 + 0.933)/2 = 0.958). The denominator for the orientation correction ratio will be the average orientation discrimination ratio (i.e., (0.822 + 0.690)/2 = 0.811). Both ratios’ numerator will be the overall average (i.e., (0.983 + 0.933 + 0.822 + 0.690)/4 = 0.857). This produces a pair of correction ratios for color-relevant discrimination ratios, 0.894 (i.e., 0.857/0.958) and orientation-relevant ratios 1.134 (i.e., 0.857/0.811) for multiplication with the corresponding, original discrimination ratio. Notice that birds’ superior performance on the color discrimination will be reduced because the correction ratio is <1 and that their inferior performance on the orientation discrimination will be boosted because its ratio is >1. This process could be repeated to create session-specific correction ratios, which would best accommodate variation in the color-orientation bias as the discrimination changes with training.

The correction ratio may also be expanded to include more than a pair of stimuli. For example, a sensory preconditioning test could include some third comparison flavor (e.g., umami), that would be counterbalanced across treatments with sucrose and saline. A third correction ratio with the denominator based on the mean uncorrected umami consumption, irrespective of stimulus role, would be used to correct the umami consumption data. The three flavors would have their own correction ratio with the overall average consumption as the numerator and the average consumption of that particular flavor as the denominator.

Where multiple nuisance variables (e.g., overall differences in performance in different operant chambers; unwanted sex differences, etc.) affect the primary measurement, multiple correction ratios can be employed. For example, if George and Pearce had found that discrimination ratios varied across their eight Skinner boxes, they could compute correction ratios for each of the eight boxes and apply them appropriately to each bird’s data. This could be done in addition the correction for color-orientation bias. In this case the box and color-orientation biases would be corrected in equal measure. Of course, the influence of the two variables is unlikely to be equal and it may be preferable to weight each set of correction ratios before their application to the original data.

It is important to note that the correction ratio will not always improve effect size. In the sensory preconditioning examples the correction ratio selectively improved effect sizes when there was a sucrose-saline bias; when there was no bias, the correction ratio produced no effect-size improvement. However, there was no circumstance in which the sensory preconditioning data produced a smaller effect size after application of the correction ratio. However, there are circumstances in which this will happen. For example, the data in Table 1 summarize an improvement in effect size when the correction ratio is applied to sensory preconditioning data that are affected by a sucrose preference, relative to saline (η_p_^2^ = .379 increases to η_p_^2^ = .493). Furthermore, if, for example, the consumption of B (saline) for the first rat of 23*g* is replaced with the value of 100 g, the effect size decreases from η_p_^2^ = .280 to η_p_^2^ = .266. These two observations demonstrate that the correction ratio acts only where there is a systematic bias and will not provide an arbitrary improvement to effect size.

## General Discussion

I examined two different ways of improving effect sizes in experimental data. One led us to examine the influence of discrimination ratios on effect size; the other used a correction ratio to offset the influence of a nuisance variable that may otherwise diminish effect size. The findings suggest that the Pfautz ratio (Pfautz et al., 1978) is preferred over the Kamin ratio (1969), which is similar to the Redhead (Redhead & Curtis, 2013; Redhead & Pearce, 1998) and Ennaceur (e.g., Ennaceur & Delacour, 1988) ratios. The correction ratio was seen to help effect size only when the nuisance variable had appreciable effect. We also saw that the application of correction ratios was general and fully expandable being applicable to variables with multiple levels and to (weighted) combinations of variables (such as differences in discrimination across stimulus dimension *and* Skinner box).

The focus on effect size is only one side of the benefits of the sensible application of ratios to experimental data. An alternative, but inextricably related, consideration is for the *N* requirements to reach a particular effect size. From a statistical point of view, larger *N*s are always favored, but increasing *N* has unwanted impact on time and on resources costs. Such concerns are especially acute in animal research where professional (e.g., American Psychological Association, 2012; The British Psychological Society, 2012), and legal (e.g., European Union, 2010; Home Office, 2013) responsibilities act to reduce the number of animals used in experimental work. Thus, the methods described here may contribute to meaningful reductions in animal requirement in experimentation in addition to improvements in effect size sensitivity.

## Supplementary Material

10.1037/xan0000143.supp

## Figures and Tables

**Table 1 tbl1:** One-Sample t-Statistics for Four Different Methods for Discrimination Ratio Computation With Varied CS (B) Rates and a Fixed Pre-CS (a) Rate

	Kamin *a*/(*a* + *b*)		Redhead *a*/(*a* + *b*)		Ennaceur (*a* − *b*)/(*a* + *b*)		Pfautz (*b* − *a*)/*b*	
CS rate	Ratio	μ	Ratio	μ	Ratio	μ	Ratio	μ
1	.0435	.5000	.9565	.5000	.9130	.0000	21.0000	.0000
8	.2667	.5000	.7333	.5000	.4667	.0000	1.7500	.0000
15	.4054	.5000	.5946	.5000	.1892	.0000	.4667	.0000
22	.5000	.5000	.5000	.5000	.0000	.0000	.0000	.0000
29	.5686	.5000	.4314	.5000	−.1373	.0000	−.2414	.0000
36	.6207	.5000	.3793	.5000	−.2414	.0000	−.3889	.0000
43	.6615	.5000	.3385	.5000	−.3231	.0000	−.4884	.0000
Effect size (η_p_^2^) =	.0847	—	.0847	—	.0847	—	.1569	—
*t*(6) =	.7450	—	.7450	—	.7450	—	1.0565	—
*p* =	.4844	—	.4844	—	.4844	—	.3314	—
Mean ratio =	.4381	—	.5619	—	.1239	—	3.1569	—
|Mean ratio − μ| =	.0619	—	.0619	—	.1239	—	3.1569	—
*Note*. CS = conditional stimulus. Four different discrimination ratios computed for seven CS rates with a fixed Pre-CS rate, 22. In the formula, *a* and *b*, respectively, refer to the CS and Pre-CS rates. The ratio value when the CS rate is equivalent to the Pre-CS rate (μ) is presented to the right of each ratio. The lower portion of the table depicts the results of a one-sample *t*-test for each of the four types of ratio with the associated effect size and *p* statistics. The mean of each set of ratios and the absolute difference between each mean and μ are presented below this.								

**Table 2 tbl2:** Raw and Transformed Consumption of Two Flavored Solutions

Flavor	A+	A+	Flavor	B−	B−
	Uncorrected data	Corrected data		Uncorrected data	Corrected data
Sucrose	2 g	1.5 g	Saline	23 g	34.8 g
Sucrose	7 g	5.2 g	Saline	22 g	33.3 g
Sucrose	11 g	8.2 g	Saline	17 g	25.7 g
Sucrose	12 g	9.0 g	Saline	22 g	33.3 g
Sucrose	14 g	10.5 g	Saline	14 g	21.2 g
Sucrose	14 g	10.5 g	Saline	15 g	22.7 g
Sucrose	25 g	18.7 g	Saline	2 g	3.0 g
Sucrose	25 g	18.7 g	Saline	9 g	13.6 g
Saline	5 g	7.6 g	Sucrose	19 g	14.2 g
Saline	0 g	.0 g	Sucrose	19 g	14.2 g
Saline	16 g	24.2 g	Sucrose	19 g	14.2 g
Saline	3 g	4.5 g	Sucrose	21 g	15.7 g
Saline	0 g	.0 g	Sucrose	22 g	16.4 g
Saline	5 g	7.6 g	Sucrose	22 g	16.4 g
Saline	1 g	1.5 g	Sucrose	22 g	16.4 g
Saline	0 g	.0 g	Sucrose	29 g	21.7 g
Saline	0 g	.0 g	Sucrose	29 g	21.7 g

Mean uncorrected sucrose consumption:	18.4 g	Mean corrected sucrose consumption:	13.7 g
Mean uncorrected saline consumption:	9.1 g	Mean corrected saline consumption:	13.7 g
Mean uncorrected overall consumption:	13.7 g	Mean corrected overall consumption:	13.7 g
Sucrose correction ratio:	13.7/18.4 = .7	Sucrose correction ratio:	13.7/13.7 = 1
Saline correction ratio:	13.7/9.1 = 1.5	Saline correction ratio:	13.7/13.7 = 1
*Note*. Data from a sensory preconditioning experiment with seventeen rats using flavored stimuli as the conditioned stimulus (A+) and control stimulus (B−). The flavors serving as A+ and B-were sucrose and saline with roles being counterbalanced across subjects. The top panel displays fluid-consumption data in grams in original, uncorrected form and in corrected form. The bottom left panel gives summary data for uncorrected consumption, which is expressed both by role (A+ vs. B−) and by flavor (sucrose versus saline). The bottom left panel also depicts calculation a correction-treatment ratio for the two flavors in which the average overall consumption is divided by the consumption of one or other of sucrose or saline. The corrected data is derived from the product of each uncorrected datum and the correction ratio for that flavor. The bottom right panel shows summary data, corresponding to that of the bottom left panel but for corrected data.			

## References

[c1] American Psychological Association (2012). Guidelines for ethical conduct in the care and use of nonhuman animals in research. Retrieved from http://www.apa.org/science/leadership/care/guidelines.aspx 10.1901/jeab.1986.45-127 PMC1348222 16812447

[c2] Bonardi C., & Jennings D. (2009). Learning about associations: Evidence for a hierarchical account of occasion setting. Journal of Experimental Psychology: Animal Behavior Processes, 35, 440–445. 10.1037/a0014019 19594289

[c3] Cumming G. (2011). Understanding the new statistics: Effect sizes, confidence intervals, and meta-analysis. London, United Kingdom: Taylor & Francis Ltd.

[c4] Ennaceur A., & Delacour J. (1988). A new one-trial test for neurobiological studies of memory in rats. 1: Behavioral data. Behavioural Brain Research, 31, 47–59. 10.1016/0166-4328(88)90157-X 3228475

[c5] European Union (2010). Directive 2010/63/EU of the European Parliament and of the Council of 22 September 2010 on the protection of animals used for scientific purposes. Retrieved from http://eur-lex.europa.eu/legal-content/EN/TXT/?qid=1469445744396&uri=CELEX:32010L0063

[c6] George D. N., & Pearce J. M. (1999). Acquired distinctiveness is controlled by stimulus relevance not correlation with reward. Journal of Experimental Psychology: Animal Behavior Processes, 25, 363–373. 10.1037/0097-7403.25.3.363

[c7] Harris J. A., Shand F. L., Carroll L. Q., & Westbrook R. F. (2004). Persistence of preference for a flavor presented in simultaneous compound with sucrose. Journal of Experimental Psychology: Animal Behavior Processes, 30, 177–189. 10.1037/0097-7403.30.3.177 15279509

[c8] Hoffman H. S., Selekman W., & Fleshler M. (1966). Stimulus aspects of aversive controls - long term effects of suppression procedures. Journal of the Experimental Analysis of Behavior, 9, 659–662. 10.1901/jeab.1966.9-659 5970388 PMC1338260

[c9] Home Office (2013). Guidance on the operation of the animals (Scientific Procedures) act 1986. Retrieved from https://www.gov.uk/government/publications/operation-of-aspa

[c10] Howell D. C. (2002). Statistical methods for psychology (5th ed.). Pacific Grove, CA: Duxbury.

[c11] Jacobs W. J., & LoLordo V. M. (1977). The sensory basis of avoidance responding in the rat: Relative dominance of auditory or visual warning signals and safety signals. Learning and Motivation, 8, 448–466. 10.1016/0023-9690(77)90045-5

[c12] Kamin L. J. (1969). Predictability, surprise, attention, and conditioning In Campbell B. A. & Church R. M. (Eds.), Punishment and aversive behavior (pp. 279–296). New York, NY: Appleton-Century-Crofts.

[c13] Kelley K. (2007). Confidence intervals for standardized effect sizes: Theory, application, and implementation. Journal of Statistical Software, 20, 1–24. 10.18637/jss.v020.i08

[c14] Lakens D. (2013). Calculating and reporting effect sizes to facilitate cumulative science: A practical primer for t-tests and ANOVAs. Frontiers in Psychology, 4, 863 10.3389/fpsyg.2013.00863 24324449 PMC3840331

[c15] Mackintosh N. J., & Little L. (1969). Intradimensional and extradimensional shift learning by pigeons. Psychonomic Science, 14, 5–6. 10.3758/BF03336395

[c16] Miller R. R., Laborda M. A., Polack C. W., & Miguez G. (2015). Comparing the context specificity of extinction and latent inhibition. Learning & Behavior, 43, 384–395. 10.3758/s13420-015-0186-x 26100525 PMC4641778

[c17] Montuori L. M., & Honey R. C. (2016). Perceptual learning with tactile stimuli in rats: Changes in the processing of a dimension. Journal of Experimental Psychology: Animal Learning and Cognition, 42, 281–289. 10.1037/xan0000104 27379718 PMC4933527

[c18] Pezze M. A., Marshall H. J., & Cassaday H. J. (2016). Potentiation rather than distraction in a trace fear conditioning procedure. Behavioural Processes, 128, 41–46. 10.1016/j.beproc.2016.04.003 27060226 PMC4906245

[c19] Pfautz P. L., Donegan N. H., & Wagner A. R. (1978). Sensory preconditioning versus protection from habituation. Journal of Experimental Psychology: Animal Behavior Processes, 4, 286–295. 10.1037/0097-7403.4.3.286 690566

[c20] Redhead E. S., & Curtis C. (2013). Common elements enhance or retard negative patterning discrimination learning depending on modality of stimuli. Learning and Motivation, 44, 46–59. 10.1016/j.lmot.2012.05.001

[c21] Redhead E. S., & Pearce J. M. (1998). Some factors that determine the influence of a stimulus that is irrelevant to a discrimination. Journal of Experimental Psychology: Animal Behavior Processes, 24, 123–135. 10.1037/0097-7403.24.2.123

[c22] Rescorla R. A., & Cunningham C. L. (1978). Within-compound flavor associations. Journal of Experimental Psychology: Animal Behavior Processes, 4, 267–275. 10.1037/0097-7403.4.3.267 690565

[c23] Robinson J., Sanderson D. J., Aggleton J. P., & Jenkins T. A. (2009). Suppression to visual, auditory, and gustatory stimuli habituates normally in rats with excitotoxic lesions of the perirhinal cortex. Behavioral Neuroscience, 123, 1238–1250. 10.1037/a0017444 20001107 PMC4231296

[c24] Robinson J. Whitt E. J., Horsley R. R., & Jones P. M. (2010). Familiarity-based stimulus generalization of conditioned suppression in rats is dependent on the perirhinal cortex. Behavioral Neuroscience, 124, 587–599. 10.1037/a0020900 20939659

[c25] Rouder J. N., Speckman P. L., Sun D., Morey R. D., & Iverson G. (2009). Bayesian t tests for accepting and rejecting the null hypothesis. Psychonomic Bulletin & Review, 16, 225–237. 10.3758/PBR.16.2.225 19293088

[c26] The British Psychological Society (2012). Guidelines for psychologists working with animals. Retrieved from http://www.bps.org.uk/system/files/images/animals_policy_statement.pdf

[c27] Urcuioli P. J., & Zentall T. R. (1986). Retrospective coding in pigeons’ delayed matching-to-sample. Journal of Experimental Psychology: Animal Behavior Processes, 12, 69–77. 10.1037/0097-7403.12.1.69 3701260

[c28] Ward-Robinson J., Coutureau E., Good M., Honey R. C., Killcross A. S., & Oswald C. J. P. (2001). Excitotoxic lesions of the hippocampus leave sensory preconditioning intact: Implications for models of hippocampal function. Behavioral Neuroscience, 115, 1357–1362. 10.1037/0735-7044.115.6.1357 11770066

[c29] Ward-Robinson J., Coutureau E., Honey R. C., & Killcross A. S. (2005). Excitotoxic lesions of the entorhinal cortex leave gustatory within-event learning intact. Behavioral Neuroscience, 119, 1131–1135. 10.1037/0735-7044.119.4.1131 16187841

[c30] Ward-Robinson J., & Hall G. (1999). The role of mediated conditioning in acquired equivalence. The Quarterly Journal of Experimental Psychology, 52, 335–350. 10.1080/027249999393031 10605393

[c31] Ward-Robinson J., Symonds M., & Hall G. (1998). Context specificity of sensory preconditioning: Implications for processes of within-event learning. Animal Learning & Behavior, 26, 225–232. 10.3758/BF03199215

[c32] Ward-Robinson J., Wilton L. A. K., Muir J. L., Honey R. C., Vann S. D., & Aggleton J. P. (2002). Sensory preconditioning in rats with lesions of the anterior thalamic nuclei: Evidence for intact nonspatial ‘relational’ processing. Behavioural Brain Research, 133, 125–133. 10.1016/S0166-4328(01)00465-X 12110445

[c33] Whitt E. J., Haselgrove M., & Robinson J. (2012). Indirect object recognition: Evidence for associative processes in recognition memory. Journal of Experimental Psychology: Animal Behavior Processes, 38, 74–83. 10.1037/a0025886 22103695

[c34] Whitt E., & Robinson J. (2013). Improved spontaneous object recognition following spaced preexposure trials: Evidence for an associative account of recognition memory. Journal of Experimental Psychology: Animal Behavior Processes, 39, 174–179. 10.1037/a0031344 23421400

