# Supplementary material for: Ratios and Effect Size
Source: J Exp Psychol Anim Learn Cogn. 2017 Aug 14;43(4):388–98. doi: 10.1037/xan0000143 (PMC5628573; doi:10.1037/xan0000143)
Supplement: Supplementary file 1 [file XAN-2017-1364_Supp_Mat.zip › Fig3_RscriptAndGraph.html]

Figure 3 code & figure


# Figure 3 code & figure

#### *Jasper Robinson*

#### *17/01/2017*

## 

```
# >>>> 
# >>>> NOTES
# >>>> 

# tetraptych.r
# This is to give a 2 x 2 tetraptych of the 4 
# types of ratios. 
# The abscissa is the mean of the a-distribution
# the ordinate is the actual, cluster of ratios
# 

#   This includes csv names that include SD and seed
# 2017 January 15
# 
# Derivations
# Kamin011216.R
# KaminRatio__BigStep_BigSD271116.R
# KaminRatio__BigStep_151116.R
# 7 normal distributions
# Uses Kamin's ratio: a/(a+b)
# a = CS rate
# b = preCS rate
# 
# Reponse rate normal distributions vary freely and COULD dip to below zero. 
# which would give freaky ratios >1 and <0 [wtf?!]
# to avoid this, mean ratios are not less than one and their SDs = .3
#
# 'range()' is used to check that no negatives values are 
# included in normal distributions. 
#
# >>>> 
# >>>> SET UP STUFF
# >>>> 

Ss = 500 # rnorm's number of rats
SD = .3 # rnorm's standard deviation
seedNo = 1 # sets random seed so that rnorm gives consistent results each run :)
set.seed(seedNo) # sets random seed so that rnorm gives consistent results each run :)
bRate = 22 # e.g., preCS rate
require(MBESS) # for peta^2 computation
```

```
## Loading required package: MBESS
```

```
# >>>> 
# >>>> OVERVIEW OF STEPS
# >>>> 

# 1. Generates normal distributions and a/(a+b) ratios. Computes some summary statistics for normal distribution and ratios
# 2. Draw 2 x 2 tetraptych

r1 = rnorm(Ss,1,SD) # normal a-rates with mean = 1 and SD = 'SD'
r8 = rnorm(Ss,8,SD)
r15 = rnorm(Ss,15,SD)
r22 = rnorm(Ss,22,SD)
r29 = rnorm(Ss,29,SD)
r36 = rnorm(Ss,36,SD)
r43 = rnorm(Ss,43,SD)

Kam_r1 = r1/(r1 + bRate) # Kamin ratio for each member of the vector
Kam_r8 = r8/(r8 + bRate)
Kam_r15 = r15/(r15 + bRate)
Kam_r22 = r22/(r22 + bRate)
Kam_r29 = r29/(r29 + bRate)
Kam_r36 = r36/(r36 + bRate)
Kam_r43 = r43/(r43 + bRate)

Red_r1 = bRate/(r1 + bRate) # Redhead ratio for each member of the vector
Red_r8 = bRate/(r8 + bRate)
Red_r15 = bRate/(r15 + bRate)
Red_r22 = bRate/(r22 + bRate)
Red_r29 = bRate/(r29 + bRate)
Red_r36 = bRate/(r36 + bRate)
Red_r43 = bRate/(r43 + bRate)

Abd_r1 = (r1 - bRate)/(r1 + bRate) # Abdul Ennaceaur ratio for each member of the vector
Abd_r8 = (r8 - bRate)/(r8 + bRate)
Abd_r15 = (r15 - bRate)/(r15 + bRate)
Abd_r22 = (r22 - bRate)/(r22 + bRate)
Abd_r29 = (r29 - bRate)/(r29 + bRate)
Abd_r36 = (r36 - bRate)/(r36 + bRate)
Abd_r43 = (r43 - bRate)/(r43 + bRate)

Pfa_r1 = (bRate - r1)/(bRate) # Pfautz ratio for each member of the vector
Pfa_r8 = (bRate - r8)/(bRate)
Pfa_r15 = (bRate - r15)/(bRate)
Pfa_r22 = (bRate - r22)/(bRate)
Pfa_r29 = (bRate - r29)/(bRate)
Pfa_r36 = (bRate - r36)/(bRate)
Pfa_r43 = (bRate - r43)/(bRate)

# This scatterplot explains the better peta^2 at the 
# elevated end of the ratios: The variability is much lower

KamRatios7 <- data.frame(Kam_r1, Kam_r8, Kam_r15, Kam_r22, Kam_r29, Kam_r36, Kam_r43)
RedRatios7 <- data.frame(Red_r1, Red_r8, Red_r15, Red_r22, Red_r29, Red_r36, Red_r43)
AbdRatios7 <- data.frame(Abd_r1, Abd_r8, Abd_r15, Abd_r22, Abd_r29, Abd_r36, Abd_r43)
PfaRatios7 <- data.frame(Pfa_r1, Pfa_r8, Pfa_r15, Pfa_r22, Pfa_r29, Pfa_r36, Pfa_r43)

require(extrafont) # loads extra non-Helvetica fonts
```

```
## Loading required package: extrafont
```

```
## Registering fonts with R
```

```
# require(extrafontdb) 
# loadfonts()
```

## Figure 3

Note that the `echo = FALSE` parameter was added to the code chunk to prevent printing of the R code that generated the plot.
