# Supplementary material for: Ratios and Effect Size
Source: J Exp Psychol Anim Learn Cogn. 2017 Aug 14;43(4):388–98. doi: 10.1037/xan0000143 (PMC5628573; doi:10.1037/xan0000143)
Supplement: Supplementary file 1 [file XAN-2017-1364_Supp_Mat.zip › Fig4_analyses.html]

JASP 


# Results

## ANOVA

| ANOVA - peta | | | | | | | | | | | | | |
| --- | --- | --- | --- | --- | --- | --- | --- | --- | --- | --- | --- | --- | --- |
| Cases | | Sum of Squares | | df | | Mean Square | | F | | p | | η² p | |
| ratioType |  | 5.689e -7 |  | 1 |  | 5.689e -7 |  | 554.9 |  | < .001 |  | 0.688 |  |
| SD |  | 3.290e -5 |  | 2 |  | 1.645e -5 |  | 16046.8 |  | < .001 |  | 0.992 |  |
| DistanceFromµ |  | 4.228e -5 |  | 5 |  | 8.455e -6 |  | 8247.9 |  | < .001 |  | 0.994 |  |
| ratioType ✻ SD |  | 2.842e -7 |  | 2 |  | 1.421e -7 |  | 138.6 |  | < .001 |  | 0.524 |  |
| ratioType ✻ DistanceFromµ |  | 3.984e -6 |  | 5 |  | 7.967e -7 |  | 777.2 |  | < .001 |  | 0.939 |  |
| SD ✻ DistanceFromµ |  | 2.110e -5 |  | 10 |  | 2.110e -6 |  | 2058.1 |  | < .001 |  | 0.988 |  |
| ratioType ✻ SD ✻ DistanceFromµ |  | 1.986e -6 |  | 10 |  | 1.986e -7 |  | 193.8 |  | < .001 |  | 0.885 |  |
| Residual |  | 2.583e -7 |  | 252 |  | 1.025e -9 |  |  |  |  |  |  |  |
|  | | | | | | | | | | | | | |
|  |  |  |  |  |  |  |  |  |  |  |  |  |  |
| --- | --- | --- | --- | --- | --- | --- | --- | --- | --- | --- | --- | --- | --- |
| *Note.*  Type III Sum of Squares | | | | | | | | | | | | | |

### Descriptives

| Descriptives - peta | | | | | | | | | | | |
| --- | --- | --- | --- | --- | --- | --- | --- | --- | --- | --- | --- |
| ratioType | | SD | | DistanceFromµ | | Mean | | SD | | N | |
| Kamin |  | 0.1 |  | mean\_1 |  | 1.000 |  | 4.493e -6 |  | 8 |  |
|  |  |  |  | mean\_15 |  | 1.000 |  | 1.019e -5 |  | 8 |  |
|  |  |  |  | mean\_29 |  | 1.000 |  | 6.471e -6 |  | 8 |  |
|  |  |  |  | mean\_36 |  | 1.000 |  | 1.434e -6 |  | 8 |  |
|  |  |  |  | mean\_43 |  | 1.000 |  | 7.569e -7 |  | 8 |  |
|  |  |  |  | mean\_8 |  | 1.000 |  | 8.313e -6 |  | 8 |  |
|  |  | 0.2 |  | mean\_1 |  | 1.000 |  | 1.792e -5 |  | 8 |  |
|  |  |  |  | mean\_15 |  | 0.999 |  | 4.014e -5 |  | 8 |  |
|  |  |  |  | mean\_29 |  | 0.999 |  | 2.547e -5 |  | 8 |  |
|  |  |  |  | mean\_36 |  | 1.000 |  | 5.769e -6 |  | 8 |  |
|  |  |  |  | mean\_43 |  | 1.000 |  | 3.027e -6 |  | 8 |  |
|  |  |  |  | mean\_8 |  | 1.000 |  | 3.327e -5 |  | 8 |  |
|  |  | 0.3 |  | mean\_1 |  | 0.999 |  | 4.020e -5 |  | 8 |  |
|  |  |  |  | mean\_15 |  | 0.997 |  | 8.891e -5 |  | 8 |  |
|  |  |  |  | mean\_29 |  | 0.999 |  | 5.639e -5 |  | 8 |  |
|  |  |  |  | mean\_36 |  | 1.000 |  | 1.306e -5 |  | 8 |  |
|  |  |  |  | mean\_43 |  | 1.000 |  | 6.809e -6 |  | 8 |  |
|  |  |  |  | mean\_8 |  | 0.999 |  | 7.490e -5 |  | 8 |  |
| Pfautz |  | 0.1 |  | mean\_1 |  | 1.000 |  | 1.236e -6 |  | 8 |  |
|  |  |  |  | mean\_15 |  | 1.000 |  | 7.231e -6 |  | 8 |  |
|  |  |  |  | mean\_29 |  | 1.000 |  | 8.703e -6 |  | 8 |  |
|  |  |  |  | mean\_36 |  | 1.000 |  | 2.479e -6 |  | 8 |  |
|  |  |  |  | mean\_43 |  | 1.000 |  | 1.653e -6 |  | 8 |  |
|  |  |  |  | mean\_8 |  | 1.000 |  | 3.857e -6 |  | 8 |  |
|  |  | 0.2 |  | mean\_1 |  | 1.000 |  | 4.962e -6 |  | 8 |  |
|  |  |  |  | mean\_15 |  | 0.999 |  | 2.862e -5 |  | 8 |  |
|  |  |  |  | mean\_29 |  | 0.999 |  | 3.427e -5 |  | 8 |  |
|  |  |  |  | mean\_36 |  | 1.000 |  | 9.928e -6 |  | 8 |  |
|  |  |  |  | mean\_43 |  | 1.000 |  | 6.610e -6 |  | 8 |  |
|  |  |  |  | mean\_8 |  | 1.000 |  | 1.541e -5 |  | 8 |  |
|  |  | 0.3 |  | mean\_1 |  | 1.000 |  | 1.121e -5 |  | 8 |  |
|  |  |  |  | mean\_15 |  | 0.998 |  | 6.370e -5 |  | 8 |  |
|  |  |  |  | mean\_29 |  | 0.998 |  | 7.586e -5 |  | 8 |  |
|  |  |  |  | mean\_36 |  | 1.000 |  | 2.236e -5 |  | 8 |  |
|  |  |  |  | mean\_43 |  | 1.000 |  | 1.487e -5 |  | 8 |  |
|  |  |  |  | mean\_8 |  | 1.000 |  | 3.463e -5 |  | 8 |  |
|  | | | | | | | | | | | |

#### Descriptives Plots

##### DistanceFromµ: mean\_1

##### DistanceFromµ: mean\_15

##### DistanceFromµ: mean\_29

##### DistanceFromµ: mean\_36

##### DistanceFromµ: mean\_43

##### DistanceFromµ: mean\_8

## Bayesian ANOVA

| Model Comparison - peta | | | | | | | | | | | |
| --- | --- | --- | --- | --- | --- | --- | --- | --- | --- | --- | --- |
| Models | | P(M) | | P(M|data) | | BF M | | BF 10 | | % error | |
| Null model |  | 0.053 |  | 6.734e -305 |  | 1.212e -303 |  | 1.000 |  |  |  |
| ratioType |  | 0.053 |  | 1.854e -305 |  | 3.337e -304 |  | 0.275 |  | 1.332e -5 |  |
| SD |  | 0.053 |  | 1.303e -283 |  | 2.346e -282 |  | 1.935e  +21 |  | 0.010 |  |
| ratioType + SD |  | 0.053 |  | 4.898e -284 |  | 8.817e -283 |  | 7.274e  +20 |  | 1.760 |  |
| ratioType + SD + ratioType  ✻  SD |  | 0.053 |  | 5.553e -285 |  | 9.995e -284 |  | 8.245e  +19 |  | 2.084 |  |
| DistanceFromµ |  | 0.053 |  | 1.037e -277 |  | 1.867e -276 |  | 1.540e  +27 |  | 0.005 |  |
| ratioType + DistanceFromµ |  | 0.053 |  | 4.649e -278 |  | 8.368e -277 |  | 6.904e  +26 |  | 2.539 |  |
| SD + DistanceFromµ |  | 0.053 |  | 1.344e -233 |  | 2.419e -232 |  | 1.995e  +71 |  | 0.879 |  |
| ratioType + SD + DistanceFromµ |  | 0.053 |  | 2.604e -233 |  | 4.687e -232 |  | 3.867e  +71 |  | 1.938 |  |
| ratioType + SD + ratioType  ✻  SD + DistanceFromµ |  | 0.053 |  | 5.827e -234 |  | 1.049e -232 |  | 8.652e  +70 |  | 2.063 |  |
| ratioType + DistanceFromµ + ratioType  ✻  DistanceFromµ |  | 0.053 |  | 6.749e -277 |  | 1.215e -275 |  | 1.002e  +28 |  | 1.321 |  |
| ratioType + SD + DistanceFromµ + ratioType  ✻  DistanceFromµ |  | 0.053 |  | 8.001e -228 |  | 1.440e -226 |  | 1.188e  +77 |  | 1.438 |  |
| ratioType + SD + ratioType  ✻  SD + DistanceFromµ + ratioType  ✻  DistanceFromµ |  | 0.053 |  | 2.209e -228 |  | 3.976e -227 |  | 3.280e  +76 |  | 2.352 |  |
| SD + DistanceFromµ + SD  ✻  DistanceFromµ |  | 0.053 |  | 6.782e -163 |  | 1.221e -161 |  | 1.007e +142 |  | 1.329 |  |
| ratioType + SD + DistanceFromµ + SD  ✻  DistanceFromµ |  | 0.053 |  | 4.434e -159 |  | 7.981e -158 |  | 6.584e +145 |  | 6.253 |  |
| ratioType + SD + ratioType  ✻  SD + DistanceFromµ + SD  ✻  DistanceFromµ |  | 0.053 |  | 4.440e -158 |  | 7.991e -157 |  | 6.593e +146 |  | 2.308 |  |
| ratioType + SD + DistanceFromµ + ratioType  ✻  DistanceFromµ + SD  ✻  DistanceFromµ |  | 0.053 |  | 4.039e -111 |  | 7.271e -110 |  | 5.998e +193 |  | 4.974 |  |
| ratioType + SD + ratioType  ✻  SD + DistanceFromµ + ratioType  ✻  DistanceFromµ + SD  ✻  DistanceFromµ |  | 0.053 |  | 2.526e -106 |  | 4.546e -105 |  | 3.751e +198 |  | 6.589 |  |
| ratioType + SD + ratioType  ✻  SD + DistanceFromµ + ratioType  ✻  DistanceFromµ + SD  ✻  DistanceFromµ + ratioType  ✻  SD  ✻  DistanceFromµ |  | 0.053 |  | 1.000 |  | 7.127e +106 |  | 1.485e +304 |  | 4.446 |  |
|  | | | | | | | | | | | |
