# Supplementary material for: Ratios and Effect Size
Source: J Exp Psychol Anim Learn Cogn. 2017 Aug 14;43(4):388–98. doi: 10.1037/xan0000143 (PMC5628573; doi:10.1037/xan0000143)
Supplement: Supplementary file 1 [file XAN-2017-1364_Supp_Mat.zip › Fig5_analyses.html]

JASP 


# Results

## ANOVA

| ANOVA - Partial Eta Squared | | | | | | | | | | | | | |
| --- | --- | --- | --- | --- | --- | --- | --- | --- | --- | --- | --- | --- | --- |
| Cases | | Sum of Squares | | df | | Mean Square | | F | | p | | η² p | |
| AboveBelow |  | 3.220e -6 |  | 1 |  | 3.220e -6 |  | 9.534 |  | 0.002 |  | 0.032 |  |
| Ratio |  | 5.689e -7 |  | 1 |  | 5.689e -7 |  | 1.684 |  | 0.195 |  | 0.006 |  |
| AboveBelow ✻ Ratio |  | 3.641e -6 |  | 1 |  | 3.641e -6 |  | 10.780 |  | 0.001 |  | 0.037 |  |
| Residual |  | 9.593e -5 |  | 284 |  | 3.378e -7 |  |  |  |  |  |  |  |
|  | | | | | | | | | | | | | |
|  |  |  |  |  |  |  |  |  |  |  |  |  |  |
| --- | --- | --- | --- | --- | --- | --- | --- | --- | --- | --- | --- | --- | --- |
| *Note.*  Type III Sum of Squares | | | | | | | | | | | | | |

### Marginal Means

| Marginal Means - AboveBelow | | | | | | | | | |
| --- | --- | --- | --- | --- | --- | --- | --- | --- | --- |
| AboveBelow | | Marginal Mean | | SE | | Lower CI | | Upper CI | |
| Above22 |  | 1.000 |  | 4.843e -5 |  | 1.000 |  | 1.000 |  |
| Below22 |  | 0.999 |  | 4.843e -5 |  | 0.999 |  | 1.000 |  |
|  | | | | | | | | | |

### Descriptives

| Descriptives - Partial Eta Squared | | | | | | | | | |
| --- | --- | --- | --- | --- | --- | --- | --- | --- | --- |
| AboveBelow | | Ratio | | Mean | | SD | | N | |
| Above22 |  | Kamin |  | 1.000 |  | 4.313e -4 |  | 72 |  |
|  |  | Pfautz |  | 1.000 |  | 5.684e -4 |  | 72 |  |
| Below22 |  | Kamin |  | 0.999 |  | 7.382e -4 |  | 72 |  |
|  |  | Pfautz |  | 1.000 |  | 5.451e -4 |  | 72 |  |
|  | | | | | | | | | |

#### Descriptives Plot

## Bayesian ANOVA

| Model Comparison - Partial Eta Squared | | | | | | | | | | | |
| --- | --- | --- | --- | --- | --- | --- | --- | --- | --- | --- | --- |
| Models | | P(M) | | P(M|data) | | BF M | | BF 10 | | % error | |
| Null model |  | 0.200 |  | 0.012 |  | 0.050 |  | 1.000 |  |  |  |
| AboveBelow |  | 0.200 |  | 0.122 |  | 0.558 |  | 9.890 |  | 6.977e -7 |  |
| Ratio |  | 0.200 |  | 0.003 |  | 0.014 |  | 0.275 |  | 1.332e -5 |  |
| AboveBelow + Ratio |  | 0.200 |  | 0.035 |  | 0.144 |  | 2.807 |  | 3.828 |  |
| AboveBelow + Ratio + AboveBelow  ✻  Ratio |  | 0.200 |  | 0.827 |  | 19.119 |  | 66.783 |  | 4.534 |  |
|  | | | | | | | | | | | |

## T-Test

| Independent Samples T-Test | | | | | | | |
| --- | --- | --- | --- | --- | --- | --- | --- |
|  | | t | | df | | p | |
| Partial Eta Squared |  | -1.258 |  | 286.0 |  | 0.209 |  |
|  | | | | | | | |
|  |  |  |  |  |  |  |  |
| --- | --- | --- | --- | --- | --- | --- | --- |
| *Note.*  Student's T-Test. | | | | | | | |

### Descriptives

#### Descriptives Plot

##### Partial Eta Squared

## Bayesian T-Test

| Bayesian Independent Samples T-Test | | | | | |
| --- | --- | --- | --- | --- | --- |
|  | | BF₁₀ | | error % | |
| Partial Eta Squared |  | 9.891 |  | 6.977e -9 |  |
|  | | | | | |
