# Supplementary material for: Ratios and Effect Size
Source: J Exp Psychol Anim Learn Cogn. 2017 Aug 14;43(4):388–98. doi: 10.1037/xan0000143 (PMC5628573; doi:10.1037/xan0000143)
Supplement: Supplementary file 1 [file XAN-2017-1364_Supp_Mat.zip › Fig2_RscriptAndGraph.html]

Figure 2 Code and figure


# Figure 2 Code and figure

#### *Jasper Robinson*

#### *17/01/2017*

## Figure 2 code

```
# >>>> 
# >>>> NOTES
# >>>> 

# Fig2-Jan16-kamin.r
# The abscissa is the mean of the a-distribution
# the ordinate is the actual, cluster of ratios
# 

# 
# Derivations
# Kamin011216.R
# KaminRatio__BigStep_BigSD271116.R
# KaminRatio__BigStep_151116.R
# 7 normal distributions
# Uses Kamin's ratio: a/(a+b)
# a = CS rate
# b = preCS rate
# 
# Reponse rate normal distributions vary freely and COULD dip to below zero. 
# which would give freaky ratios >1 and <0 [wtf?!]
# to avoid this, mean ratios are not less than one and their SDs = .3
#
# 'range()' is used to check that no negatives values are 
# included in normal distributions. 
#
# >>>> 
# >>>> SET UP STUFF
# >>>> 

Ss = 500 # rnorm's number of rats
SD = .3 # rnorm's standard deviation
seedNo = 1 # sets random seed so that rnorm gives consistent results each run :)
set.seed(seedNo) # sets random seed so that rnorm gives consistent results each run :)
bRate = 22 # e.g., preCS rate
require(MBESS) # for peta^2 computation
```

```
## Loading required package: MBESS
```

```
# >>>> 
# >>>> OVERVIEW OF STEPS
# >>>> 

# 1. Generates normal distributions and a/(a+b) ratios. Computes some summary statistics for normal distribution and ratios
# 2. Creates scatterplot

r1 = rnorm(Ss,1,SD) # normal a-rates with mean = 1 and SD = 'SD'
r8 = rnorm(Ss,8,SD)
r15 = rnorm(Ss,15,SD)
r22 = rnorm(Ss,22,SD)
r29 = rnorm(Ss,29,SD)
r36 = rnorm(Ss,36,SD)
r43 = rnorm(Ss,43,SD)

Kam_r1 = r1/(r1 + bRate) # Kamin ratio for each member of the vector
Kam_r8 = r8/(r8 + bRate)
Kam_r15 = r15/(r15 + bRate)
Kam_r22 = r22/(r22 + bRate)
Kam_r29 = r29/(r29 + bRate)
Kam_r36 = r36/(r36 + bRate)
Kam_r43 = r43/(r43 + bRate)

# This scatterplot explains the better peta^2 at the 
# elevated end of the ratios: The variability is much lower

KamRatios7 <- data.frame(Kam_r1, Kam_r8, Kam_r15, Kam_r22, Kam_r29, Kam_r36, Kam_r43)

require(extrafont) # loads extra non-Helvetica fonts
```

```
## Loading required package: extrafont
```

```
## Registering fonts with R
```

```
# require(extrafontdb) 
# loadfonts()
```

## Figure 2

Note that the `echo = FALSE` parameter was added to the code chunk to prevent printing of the R code that generated the plot.
